# Efficacy of Infection Control Interventions in Reducing the Spread of Multidrug-Resistant Organisms in the Hospital Setting

**DOI:** 10.1371/journal.pone.0030170

**Published:** 2012-02-20

**Authors:** Erika M. C. D'Agata, Mary Ann Horn, Shigui Ruan, Glenn F. Webb, Joanna R. Wares

**Affiliations:** 1 Division of Infectious Diseases, Beth Israel Deaconess Medical Center, Harvard Medical School, Boston, Massachusetts, United States of America; 2 Department of Mathematics, Vanderbilt University, Nashville, Tennessee, United States of America; 3 Division of Mathematical Sciences, National Science Foundation, Arlington, Virginia, United States of America; 4 Department of Mathematics, University of Miami, Coral Gables, Florida, United States of America; 5 Department of Math and Computer Science, University of Richmond, Richmond, Virginia, United States of America; New York State Health Department and University at Albany, United States of America

## Abstract

Multidrug-resistant organisms (MDRO) continue to spread in hospitals globally, but the population-level impact of recommended preventive strategies and the relative benefit of individual strategies targeting all MDRO in the hospital setting are unclear. To explore the dynamics of MDRO transmission in the hospital, we develop a model extending data from clinical individual-level studies to quantify the impact of hand hygiene, contact precautions, reducing antimicrobial exposure and screening surveillance cultures in decreasing the prevalence of MDRO colonization and infection. The effect of an ongoing increase in the influx of patients colonized with MDRO into the hospital setting is also quantified. We find that most recommended strategies have substantial effect in decreasing the prevalence of MDRO over time. However, screening for asymptomatic MDRO colonization among patients who are not receiving antimicrobials is of minimal value in reducing the spread of MDRO.

## Introduction

Multidrug-resistant organisms (MDRO), including methicillin-resistant *Staphylococcus aureus* (MRSA), vancomycin-resistant enterococci (VRE) and multidrug-resistant gram-negative bacteria (MDRGN), continue to spread in hospitals worldwide causing substantial morbidity and mortality [1]. Limiting the emergence and spread of MDRO requires a multifaceted approach which encompasses decreasing MDRO transmission between patients, limiting inappropriate antimicrobial exposure and reducing MDRO infections among MDRO-colonized patients [1]. The population-level impact of strategies targeting all MDRO is unclear since the great majority of clinical investigations addressing their efficacy have been individual- or patient-level studies focusing on a single MDRO pathogen.

The spread of MDRO is a complex system, with numerous interrelated and dynamic interactions between patients and healthcare workers (HCW), and therefore focusing on individuals by themselves or a single type of MDRO provides incomplete answers. Population-level models, in contrast, relate individuals to each other and have been instrumental in understanding the efficacy of preventive strategies targeting numerous infectious agents, including influenza, HIV and multidrug-resistant tuberculosis [2]–[7]. These mathematical models build upon clinical individual-level data and characterize the relationship between individuals and MDRO spread at the population level. Modeling is therefore a necessary requirement for the extension and complete analysis of epidemiological data of MDRO, since clinical epidemiological studies cannot fully capture the complexities of MDRO spread at the population level. Another inherent limitation of clinical studies of infectious agents is the assumption of independence between individuals. Since the spread of MDRO, by definition, occurs between patients, independence between individuals cannot be assumed [8]. A comprehensive transmission model was therefore developed to quantify the population-level impact of preventive strategies targeting all MDRO, including MRSA, VRE and MDRGN, using data obtained from clinical studies and the study hospital. The outcomes of interest were the overall reduction in the prevalence of MDRO colonization and MDRO infection.

### Mathematical Model

**Figure 1:**
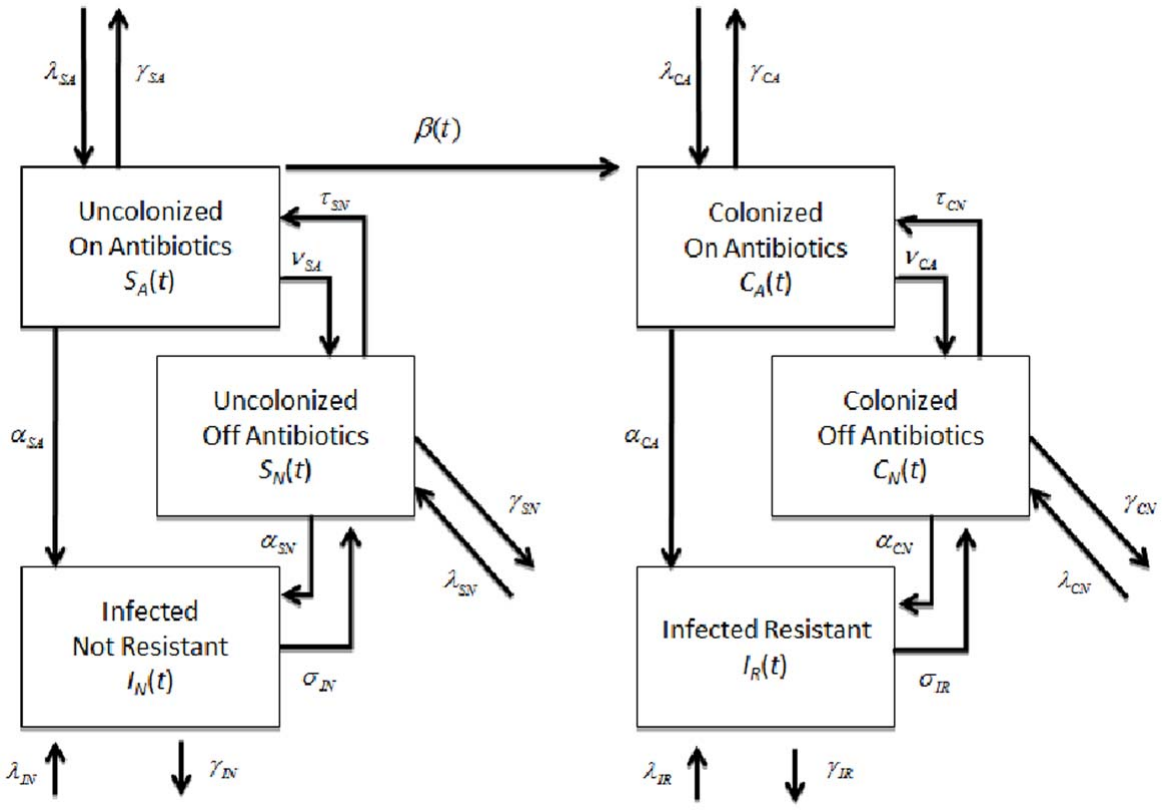
A compartmental model describing the transmission dynamics of MDROs in a 600-bed hospital. The arrows and parameter values correspond to entry and exit from the 6 compartments (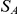-susceptible patients receiving antibiotics, 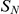-susceptible patients not receiving antibiotics, 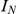-patients infected with a non-resistant strain, 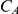-patients colonized with an MDRO that are receiving antibiotics, 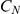-patients colonized with an MDRO not receiving antibiotics, and 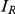-patients infected with an MDRO). The following parameters were used: 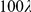, percentage of patients being admitted to each compartment; 1/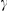, length of hospital stay; 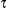, rate that patients start receiving antibiotics; 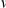-rate that patients discontinue antibiotics; 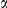, rate that colonized patients become infected; 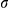, rate that infected patients are successfully treated.

A deterministic differential equations model characterizing the transmission dynamics of MDRO between patients and healthcare workers in a 600-bed tertiary care hospital in Boston, Massachusetts, with approximately 40,000 admissions per year was developed. In the model, hospitalized patients are in 6 mutually exclusive states: colonized with MDRO receiving or not receiving antimicrobials (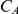 and 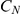 respectively), not colonized with MDRO receiving or not receiving antimicrobials (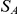 and 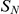, respectively), infected with antimicrobial-susceptible pathogens (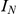) or infected with MDRO (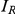). To simplify the model, the infected and colonized with non-resistant bacteria were combined. This coupling allowed direct comparisons to the resistant compartments. Patients enter the hospital in any of the 6 states and leave at a rate of 1/length of average hospital stay specific for each compartment. Uncolonized patients receiving antimicrobials become colonized with MDRO through contact with HCW and move into the MDRO-colonized, on antimicrobials, compartment. Healthcare worker interactions are implicitly modeled through variations in the transmission rates. It is assumed that only uncolonized patients receiving antimicrobials can become colonized with MDRO, since selective pressure on the patient's flora from antimicrobial exposure is a prerequisite for MDRO acquisition [9]. Although this assumption is theoretically valid, we also simulated the model allowing all patients to become colonized with the resistant strain regardless of antimicrobial exposure. The model incorporates the decreased transmission probability among colonized patients who are not receiving antimicrobials. In these patients, the absence of selective pressure from antimicrobials results in lower MDRO bacterial loads and leads to a lower likelihood of skin and environmental contamination [10]–[12]. It was also assumed that loss of MDRO colonization does not occur during a patient's hospital stay since for all MDRO, colonization exceeding the average length of hospital stay has been well-documented [13], [14]. During their hospital stay, a proportion of uncolonized and colonized patients develop infections with antimicrobial-susceptible pathogens and MDRO, respectively. Once antimicrobial treatment is completed, patients move back to their respective uncolonized or colonized, not receiving antimicrobials, compartments (Figure 1). Mathematical equations describing the transmission dynamics of MDRO are provided in the Methods section.

Contact precautions (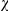), which reduce the probability of MDRO transmission (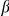), are implemented among patients who have infections with MDRO and among colonized patients identified by screening [15]. These contact precautions include donning gloves and gowns upon entering a colonized patient's room to prevent HCW contamination and subsequent MDRO spread to other patients. Distinct values for screening efficacy among patients receiving and not receiving antimicrobials were incorporated into the model (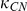 and 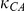), since studies have shown that an increased bacterial load due to antimicrobial exposure significantly increases the diagnostic accuracy of screening cultures [10]. Healthcare worker hand hygiene compliance (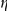) was incorporated into the model transmission rates (see Methods).

Parameter estimates for the model were obtained from the study site's extensive computerized databases of patient admissions, pharmacy, infection control and microbiological repositories from January 1st through December 31st, 2009. This real-time web-based on-line medical record system provides data for all hospitalizations to the study hospital. Estimates that were not available from these databases were obtained from a formal review of the literature (Table 1).

**Table 1:** Parameter estimates for the model of transmission dynamics of multidrug-resistant organisms (MDRO).

Parameter	Symbol	Baseline Value	Source
Percentage of patients admitted per day			
- uncolonized receiving antimicrobials	100 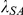	4.5%	S, [22]–[24]
- uncolonized not receiving antimicrobials	100 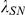	80%	
- colonized receiving antimicrobials	100 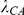	0.3%	
- colonized not receiving antimicrobials	100 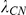	10%	
- infected with MDRO	100 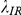	0.2%	
- infected with antimicrobial-susceptible bacteria	100 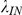	5%	
Length of hospital stay			
- uncolonized receiving antimicrobials	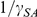	10 days	S
- uncolonized not receiving antimicrobials	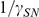	5 days	
- colonized receiving antimicrobials	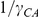	14 days	
- colonized not receiving antimicrobials	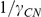	14 days	
- infected with MDRO	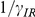	30 days	
- infected with antimicrobial-susceptible bacteria	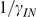	10 days	
Patients in whom antimicrobials are discontinued per day			
- colonized	100 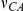	4%	S
- uncolonized	100 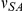	15%	
Patients in whom antimicrobials are started per day			
- colonized	100 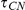	25%	S
- uncolonized	100 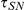	15%	
Infected cure rate			
- MDRO	100 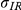	65%/LOS	[28], [29]
- non-MDRO	100 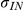	80%/LOS	
Becoming infected rate			
- colonized	100 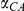 , 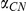	5%/LOS	[30]
- uncolonized	100 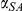 , 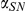	5%/LOS	[30]
Probability of HCW transmission in contact with			
- colonized patients not on contact precautions	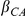	0.4	[10], [15], [31]
- patient with MDRO infection on contact precautions	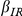	0.4 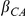	
- colonized patients not receiving antimicrobials	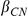	0.2 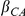	S
Compliance with hand hygiene or contact precautions	100 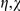	60%	[32]
Screening efficacy colonized receiving antimicrobials	100 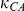	90%	
Screening efficacy colonized not receiving antimicrobials	100 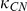	50%	

S: data was obtained from a real-time web-based on-line medical record system providing information for all hospitalizations to a 600-bed tertiary care hospital from January 1st through December 31st, 2009.

A deterministic model was used and therefore patients were aggregated into homogenous compartments. Although a stochastic, individual-based model, would consider patient heterogeneity, the increase in behavioral detail would result in data that are more difficult to interpret and apply, compared to deterministic models [16].

### Evaluation of strategies

Simulations were performed to quantify the impact of the following strategies which directly affect MDRO transmission: 1) improving compliance with hand hygiene; 2) improving compliance with contact precautions; and 3) the impact of screening for asymptomatically colonized patients among those receiving and not receiving antimicrobials. Only screening for MRSA and VRE was incorporated into the model, since there is currently no standardized method for MDRGN screening. Decolonization was not addressed in this model since effective decolonization therapy is only available for MRSA. Simulations were also performed to determine the contribution of factors which indirectly impact MDRO transmission: 1) the number of patients entering the hospital already colonized with MDRO, 2) length of hospital stay (LOS) of colonized patients and 3) reducing antimicrobial exposure. Lastly, simulations were also performed to determine the impact of different transmission rates among MDRO on the above strategies. The effect of varying the infection rate among colonized patients on the number was compared to the increase in number of infections if cross-transmission was minimized through non-compliance with preventive measures. The varying rate of infection reflected measures aimed at preventing the development of nosocomial infections which have been outlined by the Society for Healthcare Epidemiology of America [17].

## Results

### Baseline scenario

Using the baseline parameters (see Table 1), the model predicts that at steady-state, 24% of patients will be colonized with MDRO, 2.1% will develop MDRO infections, and 7% will develop non-MDRO infections. Approximately 5–10% of patients will develop an infection while in the hospital, with the upper limit representing hospitals similar to the study hospital, with a population of patients with a greater severity of illness and therefore with a higher likelihood of developing hospital-acquired infections [18]. The baseline model's estimate of a total of 9.1% of infections is therefore comparable to published data.

There are approximately ten times more MDRO-colonized patients than MDRO infected patients in the hospital setting as shown in several studies [14], [19]. The model's estimates of 24% of MDRO-colonized patients and 2.1% of MDRO infected patients are therefore also comparable to published data. Simulations which allow all patients to become colonized regardless of antimicrobial exposure, as opposed to only those receiving antimicrobials, did not change the qualitative results of the baseline model.

### Hand hygiene and contact precautions

**Figure 2:**
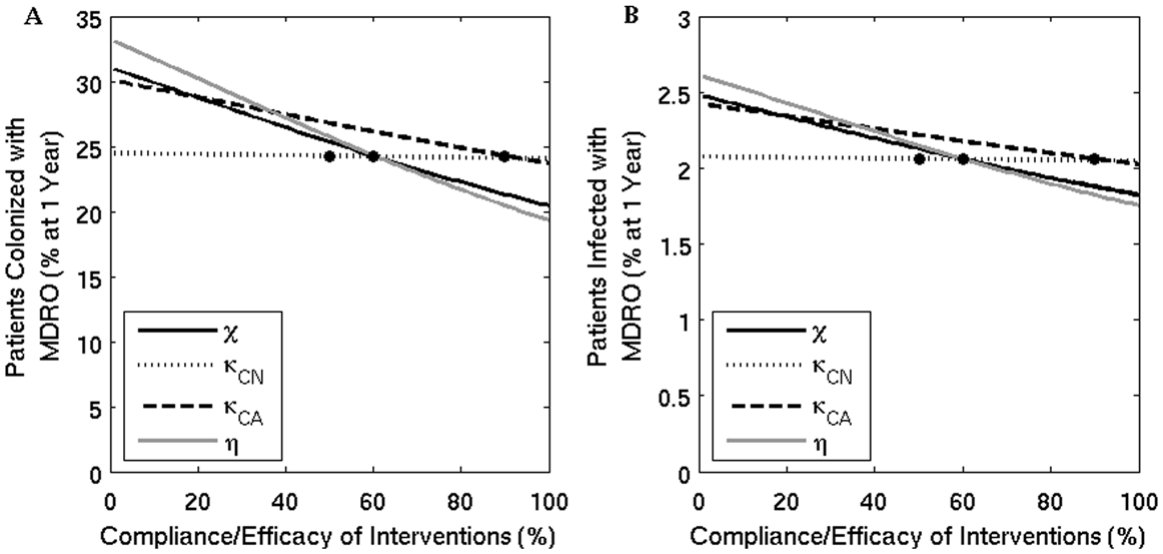
The percent of patients colonized (A) and infected (B) with an MDRO at one year when the compliance or efficacy of four interventions are varied. Solid black line (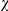) - contact precautions, dotted line (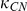) - screening of colonized patients not on antimicrobials, dashed line (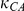) - screening of colonized patients receiving antimicrobials, and grey line (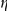) - compliance with hand hygiene measures. The dots mark the baseline values.

**Figure 3:**
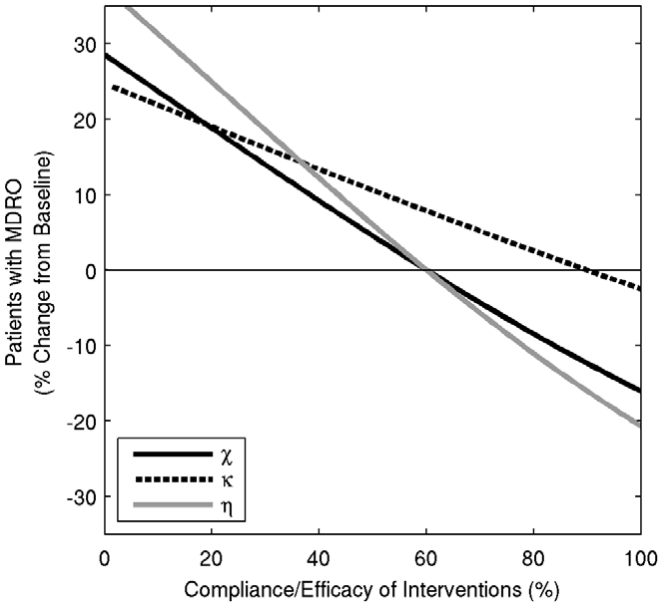
The percentage change from baseline of patients that are colonized with an MDRO at one year. Grey line - hand hygiene measure compliance is varied; solid black line - the percentage of compliance with contact precautions is varied; dashed line - the efficacy of screening patients receiving antibiotics is varied.

The estimated benefits of improving compliance with hand hygiene and contact precautions in reducing the overall percent of patients colonized or infected with MDRO are presented in Figure 2. In figure 3, the change in percent of MDRO-colonized patients from baseline values is quantified, as compliance with hand hygiene or contact precautions vary. Improvements in compliance with hand hygiene from 60% to 80% and from 80% to 100% decrease the prevalence of colonization by 12% and 8% respectively, and both decrease the percent of patients with MDRO infections by 8%. An improvement in compliance with contact precautions from 60% to 80% and from 80% to 100% decreases the prevalence of colonization by 10% and 6% respectively, and decreases the percent of patients with MDRO infections by 6% and 4%, respectively. These decreases in infection percent reflect the overall decrease in the total number of patients colonized with MDRO.

### Screening

Screening patients for asymptomatic colonization also reduces the overall prevalence of MDRO, but only among patients receiving antimicrobials (Figures 2 and 3). Improving screening efficacy has less effect on the prevalence of MDRO compared to improving compliance with hand hygiene or contact precautions, since a smaller population size is targeted and the model only incorporates screening for VRE and MRSA, since MDRGN screening has not been standardized.

### Influx into the hospital and LOS of MDRO-colonized patients

**Figure 4:**
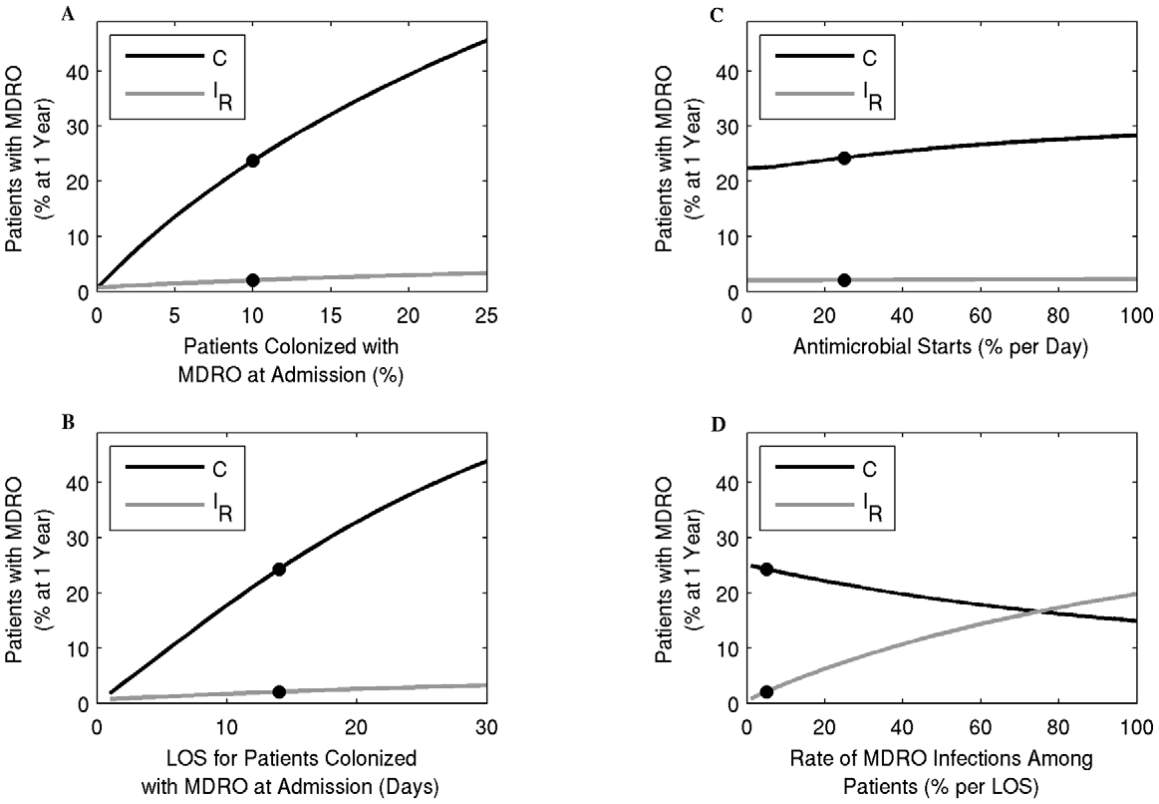
The percent of patients that are colonized with MDRO (black line) or infected with MDRO (grey line) at one year. (A) - the percentage of patients colonized with MDRO at admission is varied; (B) - the length of stay (LOS) of patients colonized with MDRO is varied; (C) - the percent of MDRO-colonized patients becoming infected during their stay in the hospital is varied; and (D) - the percent of MDRO-colonized patients starting antimicrobials per day. The dots mark the baseline values.

Increasing the influx of MDRO-colonized patients into the hospital results in a substantial increase in the prevalence of MDRO-colonized patients over time. Figure 4a shows that increasing the influx from 10% to 15% to 20% will increase the percent of MDRO-colonized patients from 24% to 32% to 40%, respectively. Similar increases in the prevalence of MDRO occur if the LOS of colonized patients increases from the baseline of 14 days to 21 to 28 days, resulting in an increase in prevalence from 24% to 34% to 42%, respectively (Figure 4b). When comparing the impact of the influx or LOS of MDRO-colonized patients to the efficacy of prevention strategies directly affecting transmission in the overall prevalence of MDRO, Figures 2a, 4a and 4b, demonstrate that increasing the influx to only 14% or LOS to 18 days results in prevalence values over 30%, which are similar to values when compliance with hand hygiene or contact precautions are 0% or there is no screening implemented.

### Antimicrobial exposure

At the study hospital, antimicrobials are initiated among 25% and 15% of MDRO-colonized and uncolonized patients per day, respectively (Table 1). Increasing the percentage of patients who start antimicrobials from 0% to 100% results in an overall increase in MDRO prevalence from 22% to 30% (Figure 4c). Decreasing antimicrobial exposure has less effect on decreasing the prevalence of MDRO compared with improving hand hygiene or contact precaution compliance, since this intervention only impacts the subset of colonized patients receiving antimicrobials.

### Varying transmission rates

**Figure 5:**
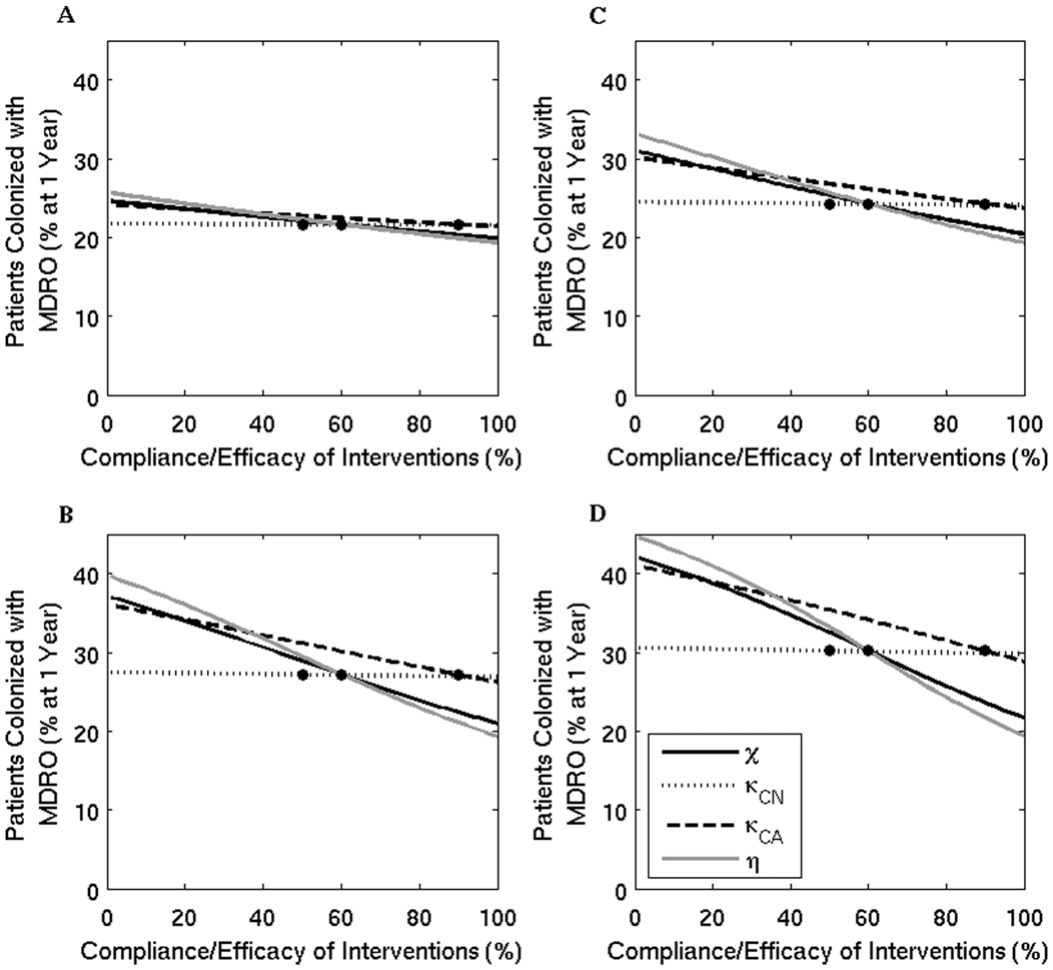
The percent of patients colonized with an MDRO at one year when the compliance or efficacy of four interventions are varied for four transmission levels. Solid black line (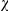) - contact precautions, dotted line (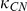) - screening of colonized patients not on antimicrobials, dashed line (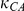) - screening of colonized patients receiving antimicrobials, and grey line (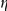) - compliance with hand hygiene measures. The dots mark the baseline values. (A) 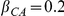; (B) 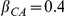 - baseline value used in other simulations; (C) 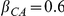; and (D) 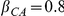.

**Figure 6:**
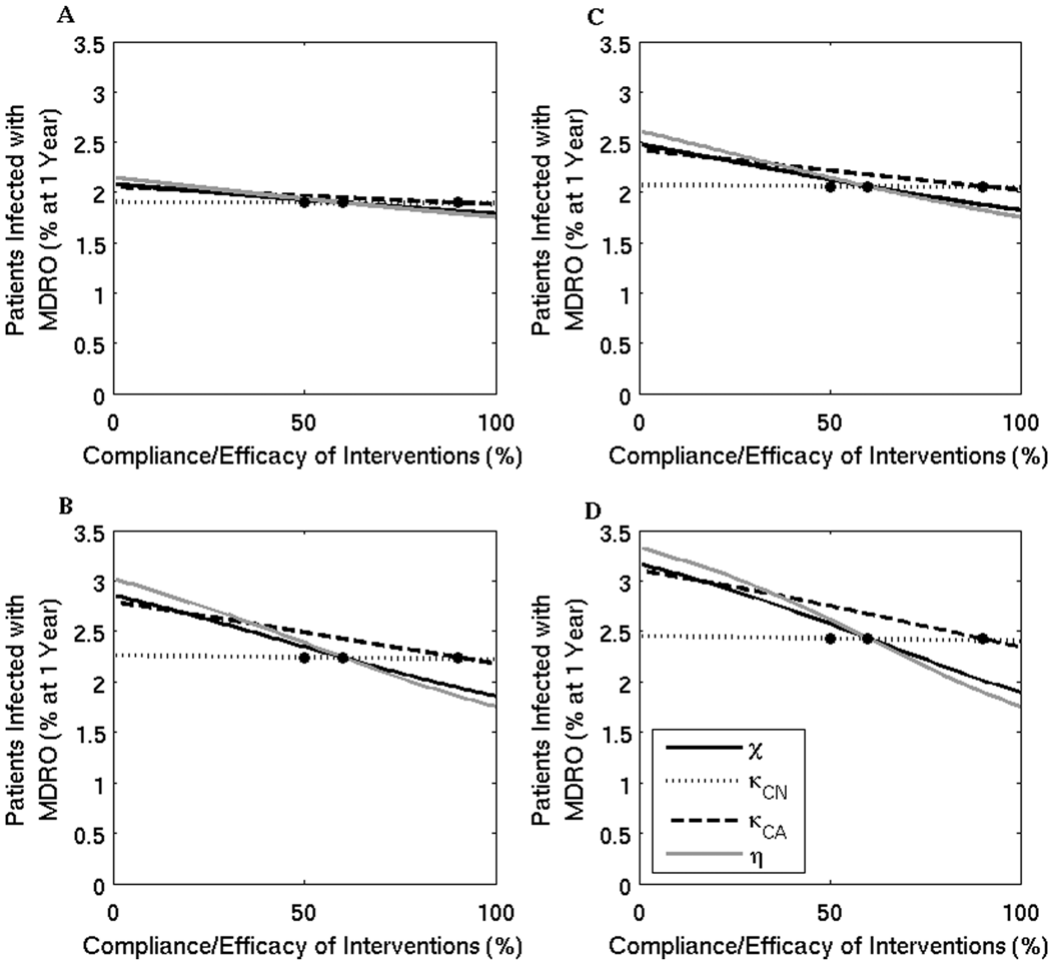
The percent of patients infected with an MDRO at one year when the compliance or efficacy of four interventions are varied for four transmission levels. Solid black line (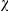) - contact precautions, dotted line (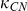) - screening of colonized patients not on antimicrobials, dashed line (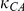) - screening of colonized patients receiving antimicrobials, and grey line (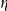) - compliance with hand hygiene measures. The dots mark the baseline values. (A) 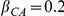; (B) 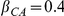 - baseline value used in other simulations; (C) 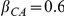; and (D) 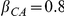.

Figure 5 and Figure 6 show the percentage of patients colonized or infected respectively, as the compliance/efficacy of contact precautions, screening patients not receiving antimicrobials, screening patients receiving antimicrobials, and hand hygiene are varied. The different panels show results for different MDRO transmission rates. These simulations show that trends remain the same, but the overall prevalence percentages are amplified for each strategy as transmission increases. Importantly, the effect of screening patients not receiving antimicrobials remains minimal, even for high transmission rates.

### MDRO infection rate among colonized patients

Varying the percent of MDRO-colonized patients who develop an infection from 5%, 10%, 15%, 20% to 25% over their LOS results in 12 (2%), 24 (4%), 33 (5.5%), 42 (7%), and 48 (8%) of hospital-acquired infections, respectively (Figure 4d). These increases in the number of infections are substantially higher than if any of the above strategies aimed at decreasing the spread of MDRO were not implemented (Figures 2b and 4a,b,c).

### Basic Reproduction Number

Here we consider the special case where all patients entering the hospital are not colonized or infected and are not receiving antimicrobials, that is 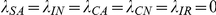 and 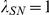. This is a model with perfect entrance screening. In this case, MDRO only remain in the hospital indefinitely due to transmission dynamics. Under this condition, we find analytic formulas for equilibria 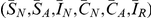 and the basic reproductive number 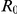.

Solving, we find two equilibria, the uncolonized equilibrium 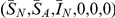 and the colonized equilibrium 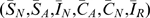 (see Methods for formulas). The uncolonized equilibrium always exists. The uncolonized equilibrium acts like the usual disease-free equilibrium of epidemic models. Correspondingly, we find the basic reproductive number, 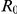. When 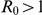, there exists another equilibrium, the colonized equilibrium, where all compartments are positive and MDRO exist in the hospital (see Methods for formulas).

The basic reproductive number for our model is given by
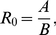
(1)where
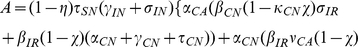


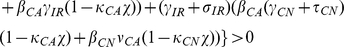


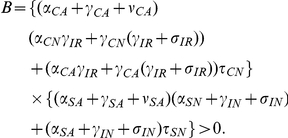



The parameters for efficacy of hand hygiene (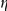) and contact precautions (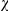) enter 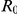 only in the numerator. When either efficacy increases, 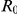 decreases and the uncolonized equilibrium is more likely to be stable. For a more complicated example, the parameters for the efficacy of screening patients also only enter 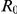 in the numerator. However, our numerical results suggest that screening patients receiving antimicrobials has a large effect on reducing colonization and infection with MDRO, whereas screening patients that are not receiving antimicrobials does not. The screening parameters (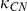 and 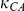) enter the numerator of 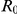 in the following products
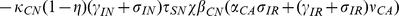


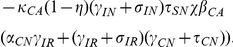



Removing all terms in common and these reduce to
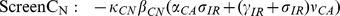


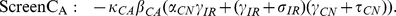



As screening efficacy increases, both terms become more negative, therefore decreasing 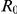. The terms are very similar, except that the magnitude of the second term is much larger due to the other terms in the product. Taking the terms in the productions one at a time, the top term, which includes screening patients that are not receiving antimicrobials (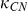), contains 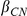 which is one-fifth the size of 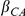, which appears in the same place in the second product. Comparing the next terms 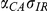 versus 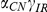, we see that 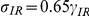 and 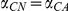, in both cases 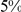 of patients that are colonized become infected while in the hospital. The final difference is between 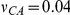 in the top equation and 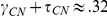 in the bottom equation. Overall, the top product has a much smaller magnitude than the bottom product, and these values are only scaled by the parameter for screening efficacy. Therefore, increasing the percentage of patients not receiving antimicrobials that are screened affects 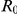 much less than increasing screening of patients that are receiving antimicrobials. This analysis mirrors our numerical results.

## Discussion

The benefits of recommended prevention strategies aimed at limiting the spread of MDRO and decreasing the rate of MDRO infections were quantified using a comprehensive transmission model. In addition to analyzing the impact of interventions which directly influence MDRO transmission, the model also quantified the impact of indirect factors which contribute to MDRO spread. In contrast to the majority of clinical studies and other models, this model addressed the transmission of all MDRO, including MRSA, VRE and MDRGN. The model also evaluated the efficacy of the most important infection control interventions and specifically addressed the differences in overall prevalence of MDRO when screening patients who were or were not receiving antimicrobials.

First, three interventions which directly reduce MDRO transmission were compared: 1) hand hygiene, 2) contact precautions, and 3) screening of asymptomatic carriers. The model projects a substantial beneficial impact in reducing the prevalence of MDRO colonization from implementation of recommended strategies. The extent of reductions in MDRO prevalence and infections reflects the proportion of patients targeted by these interventions. Hand hygiene has the most beneficial effect, as it limits transmission from all colonized patients, including those that are known and unknown to be colonized. Contact precautions are slightly less effective, since they target only those patients known to be colonized through screening or MDRO identification from clinical cultures. The model emphasizes that even modest improvements in compliance with hand hygiene or contact precautions will lead to substantial decreases in colonization. For example, the model projected that increasing compliance with hand hygiene or contact precautions from 60% to 80%, resulted in an overall decrease in the prevalence of MDRO by 10–12%.

Screening for asymptomatic MDRO colonization also reduced the prevalence of MDRO among hospitalized patients. However, screening patients who were not receiving antimicrobials had a minimal beneficial impact. The explanation for this important finding is the following. It has been well-documented that selective pressure from antimicrobials increases the MDRO bacterial load colonizing patients and that the higher bacterial load leads to greater environmental and skin contamination [10], [11]. Conversely, among patients who are not receiving antimicrobials, the absence of selective pressure results in substantially lower bacterial loads, leading to minimal environmental and skin contamination. Thus, patients not receiving antimicrobials contribute only minimally to the spread of MDRO. Screening this group of patients would therefore not have a substantial impact on reducing MDRO spread.

The finding that screening cultures performed on patients who are not receiving antimicrobials is of limited benefit, has important implications. It suggests that strategies, which implement screening cultures only on patients receiving antimicrobials may be substantially more cost-effective than those implemented on patients receiving and not receiving antimicrobials. Furthermore, although the majority of studies have shown benefits to screening cultures, the degree of benefit varies. There are numerous methodological issues which can explain the varying degree of benefit in these studies. The results of this model suggest that future studies should also evaluate the proportion of screened patients who are receiving antimicrobials [20], [21].

Second, the model evaluated three parameters, which indirectly influence the transmission rate of MDRO: 1) the percentage of patients colonized with MDRO at hospital admission, 2) the LOS among colonized patients and 3) the rate of antimicrobial exposure. Increasing the number of patients who are colonized with MDRO at admission or increasing their LOS substantially increased the prevalence of MDRO in the hospital, reflecting a larger proportion of patients transmitting MDRO for longer periods of time. Although the overall percentage of patients entering a hospital colonized with all the three MDRO under investigation has not been quantified, studies focusing on individual types of MDRO have detected up to 10%, 8% and 9% of patients harboring MRSA, VRE and MDRGN, respectively, at hospital admission, with greater percentages if admitted to the intensive care unit [22]–[27]. A proportion of these patients may be co-colonized with more than one MDRO. However these percentages emphasize the substantial influx of MDRO into the hospital setting. The model implies that the influx of MDRO into the hospital setting contributes substantially to the prevalence of MDRO in the hospital. In fact, increasing the influx of patients harboring MDRO at admission to 12% or increasing their LOS to 18 days, results in an overall prevalence of MDRO of 30% over time, a percentage value similar to when compliance with hand hygiene or contact precautions are 0% or there is no screening. An increasing reservoir of MDRO through increases in influx or LOS is also important to address when assessing the efficacy of infection control interventions. If the reservoir of MDRO increases during the study period, then the benefits of preventive strategies under study may be minimized. The third indirect factor analyzed, antimicrobial exposure, also reduced the prevalence of MDRO but less than improving compliance with hand hygiene or improving compliance with contact precautions. The smaller beneficial impact of reducing antimicrobial exposure reflects the size of the population since only colonized patients on antimicrobials are targeted.

There are two patient outcomes to consider when addressing the impact of interventions aimed at limiting MDRO transmission: colonization and infection. It is important to reduce the overall colonization prevalence in an effort to decrease the antimicrobial resistance gene pool within a healthcare setting, which contributes to endogenous acquisition of antimicrobial resistance from previously susceptible pathogens and to the emergence of novel MDRO. Preventing de novo MDRO colonization is also important as it reduces the number of patients at risk of developing infections with MDRO, once colonized. The model demonstrates that the direct interventions substantially decrease the prevalence of MDRO colonization. However, their impact on reducing MDRO infections is small, even when the efficacy of their implementation is at a maximum. In contrast, when preventing infections is the outcome of interest, even modest increases in the rate at which MDRO-colonized patients develop infections substantially negates the beneficial impact of all the strategies aimed at reducing transmission. For example, increase the MDRO infection rate among colonized patients from 5% to 10% results in as many MDRO infections as would occur if compliance with hand hygiene or contact precautions was 0%.

This model predicts qualitative trends in MDRO reduction, consistent with a wide range of baseline parameter values, which are useful for comparing the efficacy of interventions. The intention of the model is therefore not to predict precise numerical values, which can vary between hospital wards and pathogens. However, the computer programs and numerical simulations generated by this model can easily be adapted to individual hospital settings, using their specific data and support from their information systems. Simplifying assumptions of the model included the absence of environmental contamination and endogenous acquisition of antimicrobial resistance among MDRGN, which should be addressed in future models. Lastly, the majority of parameter estimates were obtained from one hospital and thus the results may not be generalizable to other healthcare settings.

This transmission model provides a comprehensive analysis of the impact of infection control interventions targeting all MDRO and extends data obtained from clinical studies by incorporating the numerous interrelated dynamic factors contributing to the spread of MDRO, which clinical studies cannot adequately address. The model also provides important findings that warrant consideration for future clinical studies addressing the impact of preventive interventions, including MDRO screening only among patients who are receiving antimicrobials, and the impact of an increasing MDRO reservoir in the hospital setting.

## Methods

The transmission of antimicrobial resistant bacteria within the hospital is modeled as a system of ordinary differential equations. The patient population is separated into six compartments: susceptible and not receiving antimicrobials (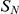), susceptible but receiving antimicrobials (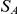), colonized with a resistant strain and not receiving antimicrobials (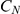), colonized with a resistant strain but receiving antimicrobials (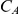), infected with a nonresistant strain (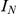), and infected with a resistant strain (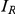). The size of each compartmental population changes in time (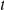), given in days, as patients transit between states. An initial size for each compartment is given at time zero (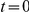).

The system of ordinary differential equations which describe transition between compartments is
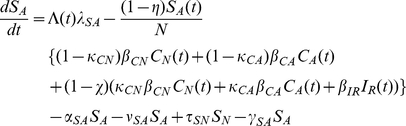
(2)

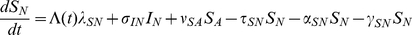
(3)

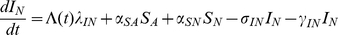
(4)

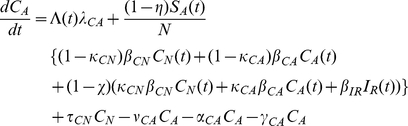
(5)

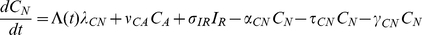
(6)

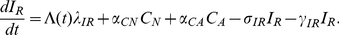
(7)


Additionally, we assume that the hospital remains full (at size 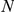) and therefore the total population size is conserved and one equation can be removed from the system by letting 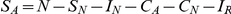. Therefore, the total rate of patients entering the hospital at any time is equal to the rate of patients leaving. The total rate at which patients leave (and therefore enter) the hospital at time 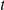 is given by
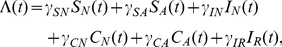
(8)where the average lengths of stay of patients are given by 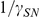, 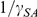, 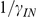, 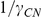, 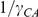, and 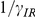 for patients in compartments 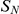, 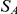, 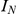, 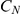, 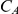 and 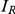 respectively.

For each parameter of the model, an independent value (demarcated by the name of the particular compartment as a subscript) is assigned to each compartment. Patients can enter the hospital in any of the six compartmental states. The proportion of patients entering susceptible and not receiving antimicrobials is 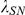, susceptible and receiving antimicrobials is 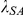, infected with a nonresistant strain is 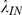, colonized and not receiving antimicrobials is 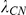, colonized and receiving antimicrobials is 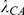 and infected with a resistant strain is 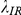. Patients in 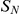, 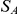, 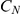, and 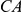 become infected at rates of 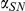, 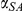, 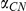 and 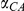, respectively. Patients infected with a resistant strain recover at a rate of 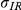 and patients infected with a nonresistant strain recover at a rate of 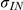. Susceptible patients begin receiving antimicrobials at a rate of 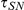, whereas colonized patients begin antimicrobials at a rate of 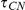. Susceptible patients stop taking antimicrobials at a rate of 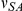, whereas, colonized patients stop antimicrobials at a rate of 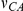.

Susceptible patients on antimicrobials become colonized with a resistant strain through the sum of mass action terms
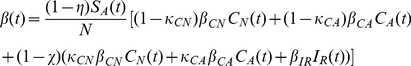
(9)where 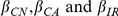 are the transmission rates for patients with a resistant strain but not on antimicrobials, patients with a resistant strain on antimicrobials, and patients infected with a resistant strain, respectively. 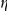 describes compliance with hand-hygiene measures; 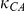 represents the efficacy of screening for patients on antimicrobials, 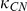 represents the efficacy of screening for patients off antimicrobials. The parameter 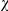 represents the percentage of compliance with contact precautions for those known to be colonized or infected with a resistant strain. Another version of the model which allowed 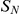 and 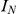 patients to become colonized with the resistant strain was also tested. These patients would become colonized through the same transmission paths as 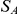 patients, but at significantly lower rates. Because of the lower rates, the results were unaffected and the simpler model was chosen for clarity.

For each set of parameters and initial conditions, a unique solution exists and remains nonnegative because the system is quasi-positive. All numerical simulations resulted in solutions that tend toward equilibria. In general, analytic formulas for the equilibria are unknown. While the entrance parameters (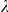) are nonzero, resistant strains will remain endemic, since there is always a source of colonized patients entering the hospital.

Consider the special case where 
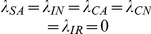
 and 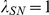. Setting all derivatives equal to zero and applying the conservation condition, the system can be written in terms of 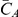 and 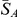:
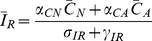
(10)

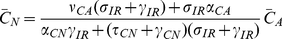
(11)

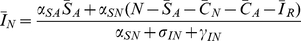
(12)

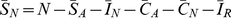
(13)

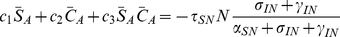
(14)

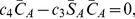
(15)where
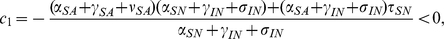


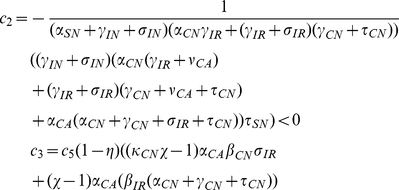


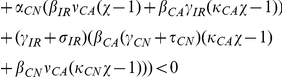


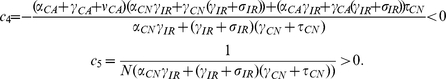



Solving, we find two equilibria, the uncolonized equilibrium 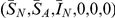 and the colonized equilibrium 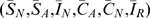. Letting 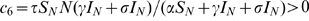, the uncolonized equilibrium is 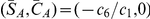. Since 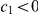, the uncolonized equilibrium always exists. Plugging these values into equations (10)–(13) gives the values for 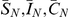 and 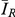. Since 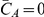, 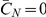 and 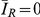. Therefore, in the uncolonized equilibrium, there are no patients colonized or infected with MDRO. The uncolonized equilibrium acts like the usual disease-free equilibrium of epidemic models. Correspondingly, we find the basic reproductive number, 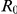. When 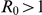, there exists another equilibrium, the colonized equilibrium, where all compartments are positive and MDRO exist in the hospital.

The colonized equilibrium has 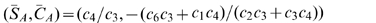. 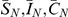 and 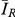 can once again be found by plugging these values into equations (10)–(13). The basic reproductive number is given in the Results section.

Simulations indicate that when 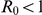, the uncolonized equilibrium is stable and the uncolonized equilibrium does not exist (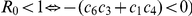. When 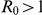, the uncolonized equilibrium is unstable and the colonized equilibrium exists and is stable (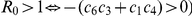. Our numerical results can be viewed through the lens of the basic reproductive number. Each parameter in the model either increases or decreases 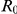. By analyzing how changes in a parameter affect 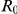, we can determine how the corresponding treatment affects the long term existence of MDRO in the hospital.

All analysis was performed using (Mathematica, Wolfram Research, Cambridge MA). Graphs were produced using MATLAB (Mathworks, Natick, MA).

## References

[1] Siegel JD, Rhinehart E, Jackson M, Chiarello L (2007). Management of multidrug-resistant organisms in health care settings.. Am J Infect Control.

[2] Temime L, Opatowski L, Pannet Y, Brun-Buisson B, Boēlle P (2009). Peripatetic health-care workers as potential superspreaders.. Proc Natl Acad Sci USA.

[3] Raboud J, Saskin R, Simor A, Loeb M, Green K (2005). Modeling transmission of methicillinresistant *Staphylococcus aureus* among patients admitted to a hospital.. Infect Control Hosp Epidemiol.

[4] Koopman JS (2004). Modeling infection transmission.. Annu Rev Public Health.

[5] Bonten M, Austin D, Lipsitch M (2001). Understanding the spread of antibiotic resistant pathogens in hospitals: mathematical models as tools for control.. Clin Infect Dis.

[6] Weinstein A, Bonten MJ, Austin DJ, Lipsitch M (2001). Understanding the spread of antibiotic resistant pathogens in hospitals: mathematical models as tools for control.. Clin Infect Dis.

[7] Sėbille V, Chevret S, Valleron A (1997). Modeling the spread of resistant nosocomial pathogens in an intensive-care unit.. Infect Control Hosp Epidemiol.

[8] Cooper M, Lipsitch M (2004). The analysis of hospital infection data using hidden Markov models.. Biostatistics.

[9] Lipsitch M, Samore M (2002). Antimicrobial use and antimicrobial resistance: a population perspective.. Emerg Infect Dis.

[10] D'Agata E, Gautam S, Green W, Tang W (2002). High rate of false-negative results of the rectal swab culture method in detection of gastrointestinal colonization with vancomycin-resistant enterococci.. Clin Infect Dis.

[11] Donskey C, Chowdhry T, Hecker MT, Hoyen C, Hanrahan J (2000). Effect of antibiotic therapy on the density of vancomycin-resistant enterococci in the stool of colonized patients.. N Engl J Med.

[12] Donskey C (2006). Antibiotic regimens and intestinal colonization with antibiotic-resistant gramnegative bacilli.. Clin Infect Dis.

[13] O'Fallon E, Gautam S, D'Agata E (2009). Colonization with multidrug-resistant gram-negative bacteria: prolonged duration and frequent cocolonization.. Clin Infect Dis.

[14] Montcalvo M, de Lencastre H, Carraher M, Gedris C, Chung M (1995). Natural history of colonization with vancomycin-resistant *Enterococcus faecium*.. Infect Control Hosp Epidemiol.

[15] Gbaguidi-Haore H, Legast S, Thouverez M, Bertrand X, Talon D (2008). Ecological study of the effectiveness of isolation precautions in the management of hospitalized patients colonized or infected with *Acinetobacter baumannii*.. Infect Control Hosp Epidemiol.

[16] D'Agata EMC, Magal P, Olivier D, Ruan S, Webb GF (2007). Modeling antibiotic resistance in hospitals: the impact of minimizing duration of treatment.. J Theor Biol.

[17] Yokoe D, Mermel L, Anderson D, Arias K, Burstin H (2008). A compendium of strategies to prevent healthcare-associated infections in acute care hospitals.. Infect Control Hosp Epidemiol.

[18] Yokoe D, Classen D (2008). Improving patient safety through infection control: a new healthcare imperative.. Infect Control Hosp Epidemiol.

[19] Ostrowsky B, Venkataraman L, D'Agata E, Gold H, DeGirolami P (1999). Vancomycinresistant enterococci in intensive care units: high frequency of stool carriage during a non-outbreak period.. Arch Intern Med.

[20] Diekmann O, Heesterbeek J, Roberts M (2010). The construction of next-generation matrices for compartmental epidemic models.. J Royal Soc Interface.

[21] Lautenbach E (2008). Expanding the universe of methicillin-resistant *Staphylococcus aureus* prevention.. Ann Intern Med.

[22] Friedmann R, Raveh D, Zartzer E, Rudensky B, Broide E (2009). Prospective evaluation of colonization with extended-spectrum beta-lactamase (ESBL)-producing enterobacteriaceae among patients at hospital admission and of subsequent colonization with esbl-producing enterobacteriaceae among patients during hospitalization.. Infect Control Hosp Epidemiol.

[23] Calderwood MS, Mauer A, Tolentino J, Flores E, van Besien K (2008). Epidemiology of vancomycin-resistant enterococci among patients on an adult stem cell transplant unit: observations from an active surveillance program.. Infect Control Hosp Epidemiol.

[24] Baker S, Brecher S, Robillard E, Strymish J, Lawler E (2010). Extranasal methicillin-resistant *Staphylococcus aureus* colonization at admission to an acute care Veterans Affairs hospital.. Infect Control Hosp Epidemiol.

[25] Ben-Ami R, Schwaber M, Navon-Venezia S, Schwartz D, Giladi M (2006). Influx of extendedspectrum beta-lactamase-producing Enterobacteriaceae into the hospital.. Clin Infect Dis.

[26] Huang S, Rifas-Shiman S, Warren D, Fraser VJ, Climo M, Wong E (2007). Improving methicillin-resistant *Staphylococcus aureus* surveillance and reporting in intensive care units.. J Infect Dis.

[27] Huang S, Rifas-Shiman S, Pottinger J, Herwaldt L, Zembower T (2007). Improving the assessment of vancomycin-resistant enterococci by routine screening.. J Infect Dis.

[28] Schwaber M, Navon-Venezia S, Kaye K, Ben-Ami R, Schwartz D (2006). Clinical and economic impact of bacteremia with extended-spectrum-beta-lactamase-producing Enterobacteriaceae.. Antimicrob Agents Chemother.

[29] Olivier C, Blake R, Steed L, Salgado C (2008). Risk of vancomycin-resistant Enterococcus (VRE) bloodstream infection among patients colonized with VRE.. Infect Control Hosp Epidemiol.

[30] Burke J (2003). Infection control - a problem for patient safety.. N Engl J Med.

[31] Austin D, Bonten M, Weinstein R, Slaughter S, Anderson R (1999). Vancomycin-resistant enterococci in intensive-care hospital settings: transmission dynamics, persistence, and the impact of infection control programs.. Proc Natl Acad Sci USA.

[32] Trick W, Vernon M, Welbel S, Demarais P, Hayden M (2007). Chicago antimicrobial resistance project. multicenter intervention program to increase adherence to hand hygiene recommendations and glove use and to reduce the incidence of antimicrobial resistance.. Infect Control Hosp Epidemiol.

